# Pubertal stage significantly and independently impacts C-peptide levels at type 1 diabetes diagnosis along with body mass index and age

**DOI:** 10.1007/s00431-025-06046-3

**Published:** 2025-03-01

**Authors:** Emine Ayça Cimbek, Nazım Ercüment Beyhun, Gülay Karagüzel

**Affiliations:** 1 Department of Pediatric Endocrinology, Faculty of Medicine, Karadeniz Technical University, Trabzon, Türkiye; 2 Department of Public Health, Faculty of Medicine, Karadeniz Technical University, Trabzon, Türkiye

**Keywords:** Type 1 diabetes, Puberty, Body mass index, Age, C-peptide, Endotypes, Children

## Abstract

Assessment of beta-cell function in type 1 diabetes (T1D) has important implications in both clinical and research settings. Studies demonstrating the extent to which puberty influences C-peptide levels are scarce. The aim of this study was to evaluate the influence of pubertal stage, along with age and body mass index (BMI), on multiple C-peptide measures at T1D diagnosis. This study included 275 consecutive children aged between 1 and 18 years with newly diagnosed T1D. Fasting, prandial, and area under the curve (AUC) C-peptide, estimated using fasting and prandial C-peptide levels, were analyzed. Generalized linear regression models were utilized. Median age at diagnosis was 7.9 (1.1–17.3) years, and mean BMI standard deviation score (SDS) was − 0.4 ± 1.4. Of the patients, 66% were prepubertal. Median fasting and prandial C-peptide levels at diagnosis were 0.26 (0.05–1.8) ng/mL and 0.43 (0.05–3) ng/mL, respectively. Fasting C-peptide was almost perfectly correlated with prandial C-peptide (r_s_ = 0.80, *P* < 0.001). Fasting, prandial, and AUC C-peptide were positively related with BMI SDS, age, and pubertal stage at diagnosis (*P* < 0.001 for all). All the associations persisted when the variables were included as independent variables in regression models. *Conclusions*: Pubertal stage significantly and independently impacts C-peptide levels at T1D diagnosis along with body mass index and age. The adjustments demonstrating the extent to which puberty influences C-peptide levels in new-onset T1D are presented. Our observations underline the existence of distinct endotypes of T1D characterized by differing immunopathological courses.

**Table:** 

**What is Known:**	
• *Throughout the evaluation of beta-cell function at T1D diagnosis, it is essential to consider the factors influencing C-peptide levels.*	
**What is New:**	
• *While age and BMI at diagnosis are associated with beta-cell function, our findings set the stage for a greater understanding of the disease process with additional findings regarding puberty, supporting the existence of endotypes of T1D.*

## Introduction

Assessing beta-cell function in type 1 diabetes (T1D) has important implications in clinical and research settings. C-peptide indices are the widely accepted measure of the efficacy of disease-modifying therapies and the primary endpoint measure in clinical trials [1, 2]. Classification schemes define T1D as a situation of insulin deficiency and type 2 diabetes as insulin resistance combined with relative insulin deficiency [3]. While current criteria of T1D diagnosis remain the presence of diabetes auto-antibodies with dysglycemia parameters [3], clinicians may use C-peptide levels to orientate toward type 1 or type 2 diabetes [4].

Throughout the evaluation of beta-cell function at T1D diagnosis, it is essential to consider the factors influencing C-peptide levels to make a precise interpretation. Although it’s known that age and body mass index (BMI) influence beta-cell function, the extent of the associations is not clear. Recently, T1D endotypes, defined by distinct pathophysiological mechanisms, have been discovered with consequences for disease prediction, prevention, diagnosis, and treatment [5]. It was shown that children < 12 years old with overweight/obesity have a higher rate of T1D progression [6]. Typically, younger age (< 7 years) is associated with a lower proportion of residual insulin-containing islets, serum C-peptide levels; higher proinsulin- to–C-peptide ratios, and frequency of diabetic ketoacidosis; hyperimmune CD20hi pattern of insulitis; HLA-DR3/DR4 haplotypes; and higher overall number of autoantibodies when compared to those diagnosed at aged ≥ 13 years [6].

It has been proposed that adjustments for BMI SDS and age could cause substantial changes in C-peptide values [7], which must be carefully interpreted as it may underline the distinct disease subgroups. Additionally, little is known regarding the relationship between C-peptide indices and pubertal status [8, 9]. The extent of the influence of puberty on C-peptide levels at T1D diagnosis is largely unknown. This study aimed to analyze the relationships between C-peptide levels and pubertal stage, along with age and BMI in children with new-onset T1D, and evaluate the extent of the associations between these variables and C-peptide indices.

## Methods

Two hundred seventy-five consecutive children aged between 1 and 18 years with T1D, newly diagnosed at the Pediatric Endocrinology Department of Karadeniz Technical University Farabi Hospital, between 2006 and 2021, participated in the study. A subset of the study population has been previously analyzed, showing the relationship between preserved C-peptide levels and age and BMI [10]. We retrospectively reviewed the medical records of the patients. T1D was diagnosed according to the International Society for Pediatric and Adolescent Diabetes guidelines, based on symptoms of insulinopenia, elevated blood glucose, and hemoglobin A1c (HbA1c) [3]. Diabetes autoantibodies levels were measured at the time of diagnosis. Insulin was started at diagnosis and used continually thereafter in all cases. Age, gender, weight, height, BMI, and pubertal stage at T1D diagnosis were recorded. BMI standard deviation scores (SDSs) were calculated according to Turkish child growth reference data using an online calculator program (http://www.childmetrics.org) [11]. Several pediatric endocrinology fellows assessed the pubertal stage according to Tanner scaling under the supervision of a faculty member, based on breast shape, the quantity and pattern of pubic hair for girls, and genital development and the quantity and pattern of pubic hair for boys. If more than one stage was detected, a mean was calculated. Laboratory findings at the time of diagnosis included C-peptide, HbA1c, pH, diabetes autoantibodies (GADA; detection limit < 1.0 U/mL), islet cell (ICA; detection limit < 2.0 U/mL), and anti-insulin (AIA; detection limit < 2.0 U/mL). Three C-peptide measures were evaluated: fasting, prandial, and area under the curve (AUC), calculated using the latter two measures. Prandial C peptide was measured 2 h after meal consumption (after restoring metabolic status, on average 2–3 days after diagnosis), and thus, it was considered stimulated.

Although the mixed-meal tolerance test (MMTT) is recognized as the gold-standard method to evaluate endogenous insulin secretion, its use in routine clinical practice is limited as it is labor-intensive and inconvenient [12]. It was shown that 90-min and 120-min MMTT C-peptide measurements were concordant with total AUC [13]. Fasting C-peptide is highly correlated with the standard MMTT and stimulated C-peptide [14]. Moreover, the assessment of C-peptide from a non-fasting random blood draw is a reasonable measure of the evaluation made during an MMTT [15]. Therefore, we determined AUC C-peptide as a function of fasting and prandial C-peptide measurements. C-peptide levels were estimated by electrochemiluminescence (ECLIA) to a sensitivity of 0.1 ng/mL. HbA1c was measured by the spectrophotometric method, and the presence of AIA, ICA, and GADA was determined by chemiluminescence immunoassay (CLIA). C-peptide values in the undetectable range were assigned a value of 0.05 ng/ml (half of the lower limit of detection) for the analyses.

IBM SPSS Statistics for Windows, version 24 (IBM Corp., Armonk, NY, USA) was used for statistical analysis. Continuous variables are expressed as mean ± standard deviation or median (min–max), and categorical variables as absolute frequency and percentage. Univariate and multivariable generalized linear regression models were used to examine associations of C-peptide variables with BMI SDS, age, and pubertal status. Models were evaluated that adjusted for variables alone and all. Pubertal stage 1 was the reference in the regression models. The significance of the model was determined by using the Omnibus test of coefficient. Normal probability distribution and identity link functions are used in the model. The significance of the regression coefficients in the model was determined by the Wald chi-square test. Pearson or Spearman correlations were also utilized. Exploratory analyses supported the use of log-transformed C-peptide measures. AUC C-peptide was calculated with the trapezoidal rule. The differences were considered statistically significant with a *P* value < 0.05.

## Results

Characteristics of the patients are shown in Table 1. Median age at diagnosis was 7.9 (1.1–17.3) years, and mean BMI standard deviation score (SDS) was − 0.4 ± 1.4. Of the patients, 66% were prepubertal and 55% were male. Median fasting and prandial C-peptide levels at diagnosis were 0.26 (0.05–1.8) ng/mL and 0.43 (0.05–3) ng/mL, respectively. Obesity (BMI ≥ 2 SDS) or overweight (BMI > 1 SDS and < 2SDS) was observed in 18%. Of the cases, 51.3% presented with DKA.

**Table 1:** Characteristics of patients

Age (years)	7.9 (1.1–17.3)
Body mass index (kg/m^2^)	16.7 ± 3.1
Body mass index SDS	− 0.4 ± 1.4
Gender (male)	151 (55%)
Puberty (prepubertal)	181 (66%)
HbA1c (%) mmol/mol	12.1 ± 2.5
	108.7 ± 27
pH	7.29 (6.8–7.5)
Diabetic ketoacidosis	141 (51.3%)
Presence of diabetes-associated autoantibodies	208 (75.6%)
Fasting C-peptide (ng/ml)	0.26 (0.05–1.8)
Prandial C-peptide (ng/ml)	0.43 (0.05–3)
AUC C-peptide (ng/ml)	0.72 (0.1–4.2)

Data presented as number (%) or mean ± standard deviation or median (min–max)

Descriptive statistics are given for each pubertal stage in Table 2. Table 3 depicts the comparisons of C-peptide indices according to the pubertal stage, showing significant differences between groups (*P* < 0.001 for all). There were increments in all indices throughout the stages on a continuum basis. Table 4 shows similar results obtained for the latter analysis after excluding autoantibody-negative patients. Subanalysis of residual C-peptide within obese and overweighted children at diagnosis compared to lean children revealed significant differences (*P* = 0.002 to 0.012) (Table 5). Another subanalysis comparing Tanner 1 with Tanner 2 and above depicted similar results (*P* < 0.001 for all) (Table 5). Reanalysis after excluding autoantibody-negative patients yielded parallel results for both subanalyses (Table 6).

**Table 2:** Characteristics of patients according to pubertal stage

Pubertal stage	1 *n* = 181	2 *n* = 40	3 *n* = 23	4 *n* = 18	5 *n* = 13
Age (years)	5.5 (1.1–12.5)	10.6 (7–13.9)	12.7 (11–14.4)	12.8 (10.3–16.5)	14.6 (12.2–17.3)
Body mass index (kg/m^2^)	15.3 (11.2–23.8)	17.8 (13.9–24.3)	18.2 (12.7–25)	17.9 (14.1–25.6)	20.3 (14.4–27.4)
Body mass index SDS	− 0.3 (− 4.4–2.5)	− 0.1 (− 2.1–2.4)	− 0.6 (− 3.86–1.91)	− 0.7 (− 3.18–1.96)	− 0.2 (− 4.9–1.72)
Gender (male)	100 (55.2%)	25 (62.5%)	13 (56.5%)	11 (61.1%)	2 (15.4%)
HbA1c (%) mmol/mol	11.8 (6.6–18.7) 105.5 (48.6–180.9)	12.2 (7.1–17.5) 109.8 (54.1–167.8)	12.6 (9.5–16.6) 114.2 (80.3–157.9)	12.8 (9.8–18.3) 116.4 (83.6–176.5)	11.3 (8.2–16.9) 100 (66.1–161.2)
pH	7.28 (6.8–7.5)	7.32 (6.9–7.42)	7.29 (7–7.37)	7.29 (7.03–7.4)	7.26 (6.83–7.4)
Diabetic ketoacidosis	95 (52.5%)	18 (45%)	12 (52.2%)	9 (50%)	7 (53.8%)
Presence of diabetes-associated autoantibodies	135 (74.6%)	33 (82.5%)	20 (87%)	12 (66.7%)	8 (61.5%)

Data presented as number (%) or mean ± standard deviation or median (min–max)

**Table 3:** Comparisons of C-peptide indices according to pubertal stage

Pubertal stage	1 *n* = 181	2 *n* = 40	3 *n* = 23	4 *n* = 18	5 *n* = 13	*P* value
Fasting C-peptide (ng/ml)	0.23 (0.05–1.76)	0.34 (0.12–1.33)	0.43 (0.13–1.52)	0.41 (0.05–1.4)	0.48 (0.17–1.25)	** < 0.001**
Prandial C-peptide (ng/ml)	0.33 (0.05–2.99)	0.62 (0.16–2.46)	0.65 (0.25–1.76)	0.69 (0.43–1.94)	0.73 (0.3–2.88)	** < 0.001**
AUC C-peptide (ng/ml)	0.56 (0.1–4.18)	1.07 (0.28–3.79)	1.09 (0.48–2.85)	1.1 (0.49–2.8)	1.17 (0.47–4.1)	** < 0.001**

Data presented as median (min–max). For the comparisons, Kruskal–Wallis test was used. Bold values indicate statistical significance at *P *< 0.05

**Table 4:** Comparisons of C-peptide indices according to pubertal stage (excluding autoantibody-negative patients)

Pubertal stage	1 *n* = 135	2 *n* = 33	3 *n* = 20	4 *n* = 12	5 *n* = 8	*P* value
Fasting C-peptide (ng/ml)	0.22 (0.05–1.76)	0.34 (0.12–1.33)	0.45 (0.13–1.52)	0.41 (0.12–1.40)	0.53 (0.17–1.25)	** < 0.001**
Prandial C-peptide (ng/ml)	0.30 (0.05–1.71)	0.73 (0.16–2.46)	0.65 (0.28–1.76)	0.69 (0.43–1.59)	0.72 (0.30–2.88)	** < 0.001**
AUC C-peptide (ng/ml)	0.50 (0.10–2.68)	1.14 (0.28–3.79)	1.09 (0.53–2.85)	1.01 (0.55–2.41)	1.10 (0.47–4.10)	** < 0.001**

Data presented as median (min–max). For the comparisons, Kruskal–Wallis test was used. Bold values indicate statistical significance at *P* < 0.05

**Table 5:** Comparisons of C-peptide indices according to weight status and pubertal status

Weight status	Lean *n* = 225	Overweight and obese *n* = 50	*P* value	Pubertal status	Pre-pubertal *n* = 181	Pubertal *n* = 94	*P* value
Fasting C-peptide (ng/ml)	0.24 (0.22)	0.45 (0.35)	**0.002**		0.22 (0.17)	0.37 (0.46)	** < 0.001**
Prandial C-peptide (ng/ml)	0.41 (0.43)	0.57 (0.56)	**0.012**		0.33 (0.33)	0.68 (0.57)	** < 0.001**
AUC C-peptide (ng/ml)	0.65 (0.68)	1.05 (0.99)	**0.004**		0.56 (0.51)	1.1 (1.1)	** < 0.001**

Data presented as median (IQR). For the comparisons, Mann–Whitney *U* test test was used. Bold values indicate statistical significance at *P* < 0.05

**Table 6:** Comparisons of C-peptide indices according to weight status and pubertal status (excluding autoantibody-negative patients)

Weight status	Lean *n* = 168	Overweight and obese *n* = 40	*P* value	Pubertal status	Pre-pubertal *n* = 135	Pubertal *n* = 73	*P* value
Fasting C-peptide (ng/ml)	0.24 (0.23)	0.37 (0.39)	**0.004**		0.22 (0.17)	0.37 (0.44)	** < 0.001**
Prandial C-peptide (ng/ml)	0.40 (0.44)	0.58 (0.68)	**0.004**		0.30 (0.31)	0.69 (0.56)	** < 0.001**
AUC C-peptide (ng/ml)	0.63 (0.65)	1.05 (1.11)	**0.002**		0.50 (0.44)	1.09 (1.08)	** < 0.001**

Data presented as median (IQR). For the comparisons, Mann–Whitney *U* test test was used. Bold values indicate statistical significance at *P* < 0.05

We observed that fasting C-peptide was almost perfectly correlated with prandial C-peptide (*r*_s_ = 0.80, *P* < 0.001). There was a positive correlation between fasting and prandial C-peptide levels and both age (*r*_s_ = 0.38, *P* < 0.001 and *r*_s_ = 0.42, *P* < 0.001, respectively) and BMI SDS (*r*_s_ = 0.27, *P* < 0.001 and *r*_s_ = 0.23, *P* = 0.001, respectively). Fasting and prandial C-peptide were also positively correlated with pubertal stage (*r*_s_ = 0.39, *P* < 0.001 and *r*_s_ = 0.44, *P* < 0.001, respectively).

Associations of fasting C-peptide, prandial C-peptide, and AUC C-peptide with age, BMI SDS, and pubertal stage are shown in Tables 7, 8, 9, and 10 that present regression models. Fasting, prandial, and AUC C-peptide levels were significantly related to age, BMI SDS, and pubertal stage at diagnosis (*P* < 0.001 for all). The R^2^ for univariate associations of C-peptide indices with BMI SDS, age, and pubertal stage ranged from 0.06 to 0.07, from 0.12 to 0.15, and 0.13 to 0.17, respectively (Table 7). Excluding autoantibody-negative patients from the analysis yielded similar results (Table 8).

**Table 7:** Univariate associations of C-peptide indices with pubertal stage, BMI SDS, and age

	BMI SDS	Pubertal stage	Age
Fasting C-peptide (ng/ml)	0.06 ± 0.01	0.1 ± 0.02	0.03 ± 0.01
	(0.06)	(0.12)	(0.13)
	***P* < 0.001**	***P* < 0.001**	***P* < 0.001**
Prandial C-peptide (ng/ml)	0.06 ± 0.02	0.1 ± 0.02	0.03 ± 0.01
	(0.06)	(0.15)	(0.15)
	***P* < 0.001**	***P* < 0.001**	***P* < 0.001**
AUC C-peptide (ng/ml)	0.06 ± 0.14	0.1 ± 0.02	0.03 ± 0.01
	(0.07)	(0.15)	(0.17)
	***P* < 0.001**	***P* < 0.001**	***P* < 0.001**

Regression coefficients, *R*^2^ (in parentheses), and *P* values are presented

Prandial C-peptide measurement was available for 204 patients

Log-transformed C-peptide measures are used. Bold values indicate statistical significance at *P* < 0.05

**Table 8:** Univariate associations of C-peptide indices with pubertal stage, BMI SDS, and age (excluding autoantibody-negative patients)

	BMI SDS	Pubertal stage	Age
Fasting C-peptide (ng/ml)	0.07 ± 0.02	0.12 ± 0.02	0.03 ± 0.01
	(0.07)	(0.14)	(0.15)
	***P* < 0.001**	***P* < 0.001**	***P* < 0.001**
Prandial C-peptide (ng/ml)	0.07 ± 0.02	0.12 ± 0.02	0.04 ± 0.01
	(0.08)	(0.17)	(0.19)
	***P* < 0.001**	***P* < 0.001**	***P* < 0.001**
AUC C-peptide (ng/ml)	0.07 ± 0.02	0.12 ± 0.02	0.04 ± 0.01
	(0.10)	(0.16)	(0.20)
	***P* < 0.001**	***P* < 0.001**	***P* < 0.001**

Regression coefficients, *R*^2^ (in parentheses), and *P* values are presented

Prandial C-peptide measurement was available for 154 patients

Log-transformed C-peptide measures are used. Bold values indicate statistical significance at *P* < 0.05

**Table 9:** Multivariate associations of C-peptide indices

	BMI SDS	Pubertal stage 5	Pubertal stage 4	Pubertal stage 3	Pubertal stage 2	Age	DKA mild	DKA moderate	DKA severe	HbA1c	Diabetes autoab
Fasting C-peptide (ng/ml)	0.05 ± 0.01 ***P* < 0.001**	0.2 ± 0.1 *P* = 0.052	0.16 ± 0.09 *P* = 0.057	0.23 ± 0.08 ***P* = 0.003**	0.09 ± 0.06 *P* = 0.106	0.02 ± 0.01 ***P* = 0.029**	− 0.13 ± 0.04 ***P* = 0.002**	− 0.16 ± 0.05 ***P* = 0.003**	− 0.2 ± 0.05 ***P* < 0.001**	− 0.03 ± 0.01 ***P* < 0.001**	− 0.1 ± 0.04 ***P* = 0.047**
Prandial C-peptide (ng/ml)	0.05 ± 0.01 ***P* < 0.001**	0.22 ± 0.11 *P* = 0.053	0.25 ± 0.09 ***P* = 0.004**	0.23 ± 0.09 ***P* = 0.009**	0.14 ± 0.06 ***P* = 0.026**	0.01 ± 0.01 *P* = 0.101	− 0.17 ± 0.05 ***P* < 0.001**	− 0.2 ± 0.06 ***P* = 0.001**	− 0.2 ± 0.05 ***P* < 0.001**	− 0.02 ± 0.01 ***P* = 0.018**	− 0.1 ± 0.04 ***P* = 0.021**
AUC C-peptide (ng/ml)	0.05 ± 0.01 ***P* < 0.001**	0.18 ± 0.1 *P* = 0.08	0.21 ± 0.08 ***P* = 0.011**	0.25 ± 0.08 ***P* = 0.002**	0.12 ± 0.06 ***P* = 0.036**	0.02 ± 0.01 ***P* = 0.042**	− 0.16 ± 0.04 ***P* < 0.001**	− 0.17 ± 0.05 ***P* = 0.001**	− 0.2 ± 0.05 ***P* < 0.001**	− 0.02 ± 0.01 ***P* = 0.004**	− 0.1 ± 0.04 ***P* = 0.013**

Regression coefficients and *P* values are presented. Prandial C-peptide measurement was available for 204 patients. Log-transformed C-peptide measures are used. Bold values indicate statistical significance at *P* < 0.05

*BMI* body mass index, *DKA* diabetic ketoacidosis

**Table 10:** Multivariate associations of C-peptide indices (excluding autoantibody-negative patients)

	BMI SDS	Pubertal stage 5	Pubertal stage 4	Pubertal stage 3	Pubertal stage 2	Age	DKA mild	DKA moderate	DKA severe	HbA1c
Fasting C-peptide (ng/ml)	0.05 ± 0.02 ***P* = 0.002**	0.16 ± 0.13 *P* = 0.236	0.19 ± 0.11 *P* = 0.079	0.25 ± 0.09 ***P* = 0.005**	0.11 ± 0.07 *P* = 0.135	0.02 ± 0.01 ***P* = 0.047**	− 0.22 ± 0.06 ***P* < 0.001**	− 0.20 ± 0.06 ***P* = 0.002**	− 0.13 ± 0.05 ***P* = 0.009**	− 0.03 ± 0.01 ***P* = 0.006**
Prandial C-peptide (ng/ml)	0.05 ± 0.02 ***P* = 0.002**	0.18 ± 0.13 *P* = 0.175	0.25 ± 0.11 ***P* = 0.019**	0.27 ± 0.10 ***P* = 0.006**	0.17 ± 0.07 ***P* = 0.019**	0.01 ± 0.01 *P* = 0.167	− 0.23 ± 0.06 ***P* < 0.001**	− 0.20 ± 0.07 ***P* = 0.003**	− 0.18 ± 0.05 ***P* = 0.001**	− 0.01 ± 0.01 *P* = 0.324
AUC C-peptide (ng/ml)	0.05 ± 0.02 ***P* < 0.001**	0.14 ± 0.13 *P* = 0.286	0.20 ± 0.10 ***P* = 0.042**	0.28 ± 0.09 ***P* = 0.002**	0.15 ± 0.07 ***P* = 0.033**	0.01 ± 0.01 *P* = 0.080	− 0.24 ± 0.06 ***P* < 0.001**	− 0.19 ± 0.06 ***P* = 0.004**	− 0.18 ± 0.05 ***P* = 0.001**	− 0.01 ± 0.01 *P* = 0.129

Regression coefficients and *P* values are presented. Prandial C-peptide measurement was available for 154 patients. Log-transformed C-peptide measures are used. Bold values indicate statistical significance at *P* < 0.05

*BMI* body mass index, *DKA* diabetic ketoacidosis

Furthermore, all the associations persisted when the variables were included as independent variables in regression models (Table 9). All C-peptide indices’ regression coefficients for BMI SDS were 0.05 ± 0.01 (*P* < 0.001). Regression coefficients were 0.23 ± 0.08 (*P* = 0.003) for fasting C-peptide and Tanner stage 3; 0.25 ± 0.09 (*P* = 0.004), 0.23 ± 0.09 (*P* = 0.009), and 0.14 ± 0.06 (*P* = 0.026) for prandial C-peptide and Tanner stages 4, 3, and 2 respectively. There was a similar relationship between AUC C-peptide and Tanner stages 4, 3, and 2. Fasting C-peptide and AUC C-peptide were significantly associated with age (regression coefficients, 0.02 ± 0.01 for both, *P* = 0.029 and 0.042, respectively). Excluding autoantibody-negative patients from the analysis did not essentially alter the results (Table 10).

Analyses were repeated after replacing BMI measured at diagnosis with BMI measured at a median of 2.7 months after diagnosis. This modification, leading to a major decrease in the number of patients, did not essentially change the results for most of the analyzed parameters (except age) (Table 11). Table 12 presents the results for the latter analysis after excluding autoantibody-negative patients.

**Table 11:** Multivariate associations of C-peptide indices (reanalysis with BMI SDS at first follow-up)

	BMI SDS first follow-up	Pubertal stage 5	Pubertal stage 4	Pubertal stage 3	Pubertal stage 2	Age	DKA mild	DKA moderate	DKA severe	HbA1c	Diabetes autoab
Fasting C-peptide (ng/ml)	0.03 ± 0.02 *P* = 0.065	0.25 ± 0.12 ***P* = 0.046**	0.19 ± 0.11 *P* = 0.085	0.23 ± 0.09 ***P* = 0.014**	0.15 ± 0.07 ***P* = 0.034**	0.01 ± 0.01 *P* = 0.113	− 0.13 ± 0.05 ***P* = 0.009**	− 0.20 ± 0.07 ***P* = 0.003**	− 0.22 ± 0.06 ***P* < 0.001**	− 0.03 ± 0.01 ***P* = 0.001**	− **0.03 ± 0.05** *P* = 0.536
Prandial C-peptide (ng/ml)	0.06 ± 0.02 ***P* = 0.004**	0.23 ± 0.14 *P* = 0.093	0.23 ± 0.11 ***P* = 0.035**	0.15 ± 0.10 *P* = 0.154	0.18 ± 0.07 ***P* = 0.013**	0.01 ± 0.01 *P* = 0.120	− 0.24 ± 0.06 ***P* < 0.001**	− 0.34 ± 0.07 ***P* < 0.001**	− 0.23 ± 0.06 ***P* < 0.001**	− 0.02 ± 0.01 *P* = 0.075	− 0.10 ± 0.05 ***P* = 0.036**
AUC C-peptide (ng/ml)	0.06 ± 0.02 ***P* = 0.002**	0.21 ± 0.143 *P* = 0.099	0.19 ± 0.10 *P* = 0.053	0.21 ± 0.10 ***P* = 0.033**	0.17 ± 0.07 ***P* = 0.011**	0.02 ± 0.01 *P* = 0.088	− 0.22 ± 0.05 ***P* < 0.001**	− 0.29 ± 0.07 ***P* < 0.001**	− 0.24 ± 0.06 ***P* < 0.001**	− 0.02 ± 0.01 ***P* = 0.028**	− 0.09 ± 0.05 ***P* = 0.045**

Regression coefficients and *P* values are presented. Fasting C-peptide was available for 193 patients and prandial C-peptide measurement for 144 patients. Log-transformed C-peptide measures are used. Bold values indicate statistical significance at *P* < 0.05

*BMI* body mass index, *DKA* diabetic ketoacidosis

**Table 12:** Multivariate associations of C-peptide indices (reanalysis with BMI SDS at first follow-up) (excluding autoantibody-negative patients)

	BMI SDS first follow-up	Pubertal stage 5	Pubertal stage 4	Pubertal stage 3	Pubertal stage 2	Age	DKA mild	DKA moderate	DKA severe	HbA1c
Fasting C-peptide (ng/ml)	0.01 ± 0.02 *P* = 0.543	0.26 ± 0.15 *P* = 0.094	0.23 ± 0.14 *P* = 0.089	0.25 ± 0.11 ***P* = 0.017**	0.17 ± 0.08 ***P* = 0.035**	0.01 ± 0.01 *P* = 0.218	−0.26 ± 0.07 ***P* < 0.001**	−0.25 ± 0.08 ***P* = 0.001**	−0.14 ± 0.06 ***P* = 0.028**	−0.03 ± 0.01 ***P* = 0.012**
Prandial C-peptide (ng/ml)	0.07 ± 0.03 ***P* = 0.006**	0.29 ± 0.16 *P* = 0.074	0.23 ± 0.13 *P* = 0.088	0.21 ± 0.12 *P* = 0.066	0.23 ± 0.08 ***P* = 0.006**	0.01 ± 0.01 *P* = 0.295	−0.24 ± 0.07 ***P* = 0.001**	−0.39 ± 0.09 ***P* < 0.001**	−0.27 ± 0.07 ***P* < 0.001**	−0.004 ± 0.01 *P* = 0.722
AUC C-peptide (ng/ml)	0.06 ± 0.02 ***P* = 0.009**	0.26 ± 0.15 *P* = 0.085	0.19 ± 0.12 *P* = 0.122	0.26 ± 0.11 ***P* = 0.015**	0.22 ± 0.08 ***P* = 0.006**	0.01 ± 0.01 *P* = 0.247	−0.26 ± 0.07 ***P* < 0.001**	−0.34 ± 0.08 ***P* < 0.001**	−0.26 ± 0.06 ***P* < 0.001**	−0.01 ± 0.01 *P* = 0.412

Regression coefficients and p values are presented. Fasting C-peptide was available for 143 patients and prandial C-peptide measurement for 105 patients. Log-transformed C-peptide measures are used. Bold values indicate statistical significance at *P* < 0.05

*BMI* body mass index, *DKA* diabetic ketoacidosis

## Discussion

These data demonstrate that an appreciable proportion of the variance of the C-peptide measures at T1D diagnosis is explained by age, BMI SDS, and pubertal stage in children with new-onset T1D, and these variables independently impact C-peptide levels at T1D diagnosis. Recent studies have reported the relationship between C-peptide levels at diagnosis and both age and the degree of adiposity [7, 16, 17]. However, most studies did not examine or take into account the effect of puberty while analyzing C-peptide levels. Moreover, in most cases, the pubertal stage was determined based on patients’ self-assessments, or age groups were used as a proxy. Recently, T1D endotypes have been discovered based on demographic, genetic, immunological, histopathological, metabolic, and/or clinical characteristics [5]. The differences in age and puberty regarding C-peptide levels shown in the current study may underline the distinct physiopathology (i.e., endotypes), and thus, we should be careful if we apply a correction of C-peptide levels according to age and/or Tanner stages. Indeed, age stratification might be more appropriate than age adjustment [7].

The heterogeneity of T1D is a significant challenge for diabetes research with high response variability observed in intervention trials. Age serves as a proxy for differences in immune and metabolic functions in T1D. Younger age is associated with higher proportions of disease progression, along with differing histological, immune, and genetic characteristics [5, 6]. Disease manifestation at an early age can be evaluated as a sign of a more aggressive autoimmune process and progression in this age group, suggesting a significant loss in insulin secretion [18, 19]. Yuan et al. [14] found a 22% increase in serum AUC C-peptide with every 1-year increase in age at diagnosis. A profound impact of advancing age at diagnosis, which was linear throughout the complete range (from 1 to 20 years), on the preservation of C-peptide levels was demonstrated among SEARCH participants [20]. Pecheur et al. [21] showed a positive effect of age on postprandial C-peptide levels in children with T1D at diagnosis and 2 years after, partly explained by differences in the treatment modalities.

Sosenko et al. [7] depicted the impact of BMI SDS and age on C-peptide indices from OGTTs at diagnosis in children with T1D. However, the authors did not determine the association between C-peptide measures and puberty. Redondo et al. [16] showed that age and BMI were associated with C-peptide levels, but pubertal stage was not independently associated with C-peptide levels at diagnosis of autoimmune T1D. That study was conducted in an ethnically diverse population, which is opposite to the present study.

A higher BMI at diagnosis is not beneficial for the long-term preservation of residual beta cell function, and this observation must also be considered in prevention studies. Steck et al. [22] showed that younger age at diagnosis, higher weight z-score (BMI SDS only in the univariate analysis), and HbA1c were associated with a steeper C-peptide decline during the two years after T1D diagnosis in young children. In another study confirming the positive association between BMI SDS and C-peptide levels only at diagnosis, C-peptide levels of obese patients dropped more rapidly in the follow-up than in children with BMI-SDS within normal limits [23]. These patients had enhanced levels of inflammatory cytokines. Weight gain contributes to insulin resistance characterized by a compensatory increase of secretory demand in beta-cells, accelerating the rate of beta-cell loss [24]. The authors emphasized that the lowest BMI-SDS group showed almost the same decline in C-peptide levels as the BMI SDS > 1 group, which means that the leanest children do not preserve residual beta-cell, too. Of note, puberty was not considered in these studies. Also, a major limitation of studying whether age of onset declines with higher BMI is that, in the general pediatric population, the prevalence of obesity increases with age, which may obscure an inverse relationship in children with T1D, if it exists [16].

Our data suggest that pubertal stage, which is intertwined with age at T1D diagnosis, is an essential factor in the disease process. Lauria et al. [25] showed that BMI is an important driver of beta-cell loss in T1D upon diagnosis only in those individuals aged 10–18 years. The authors supposed that pubertal transition, which is a process of evolution from childhood to adult reproductive capacity, ruled by the reactivation of the hypothalamic–pituitary–gonadal axis [26], explained the detrimental association of BMI with fasting C-peptide decline. Indeed, the differences in C-peptide levels between prepubertal and pubertal patients might be mostly related to different physiopathology rather than transition or insulin resistance.

Our study has several limitations and strengths. Although BMI was recorded on the day of diagnosis, results were mostly (except age, which might be due to the major decrease in the number of patients) verified when performing the same analysis with the weight at the first outpatient consultation to address the effect of dehydration upon BMI. In the previous study, which proposed that adjustments for BMI SDS and age could affect C-peptide values, the associations were not indicative of those at the time of a “clinical” T1D diagnosis [7]. Trained healthcare providers assessed the pubertal staging examination, unlike most previous studies in which patients reported their pubertal status. At an initial glance, one can say that the regression coefficients in our study are low when compared to the previous study by Sosenko et al. [7]. However, since the analyses in our study belong to the time of a clinical diagnosis, it should be considered that the average C-peptide levels are also lower, and log-transformed C-peptide measures are used. We could not measure C-peptide levels during an MMTT, as described in detail in the methods section. Although the number of patients decreased with the advancing pubertal stage, the cohort was consecutive. A limitation of the study was the relatively large number of autoantibody-negative children and adolescents with T1D in this cohort. These patients were indistinguishable from the autoantibody-positive group by clinical characteristics and did not demonstrate features typical for other diabetes subtypes.

To conclude, while age and BMI at diagnosis are key variables associated with beta-cell function, our data set sets the stage for a greater understanding of the disease process with additional findings regarding puberty. Our findings emphasize the association between the heterogeneity of T1D/ C-peptide levels and these variables, supporting the existence of endotypes of T1D. Uncovering the nature of these relationships will help in the search for effective individualized therapies.

## Data Availability

Data is provided within the manuscript.

## Abbreviations

AIA: Anti-insulin antibodies
AUC: Area under the curve
BMI: Body mass index
ECLIA: Electrochemiluminescence immune assay
GADA: Glutamic acid decarboxylase antibodies
HbA1c: Hemoglobin A1c
ICA: Islet cell antibodies
MMTT: Mixed meal tolerance test
SDS: Standard deviation score
T1D: Type 1 diabetes

## Code availability

N/A.

## Declarations

### Permission to reproduce material from other sources

N/A
